# New Jersey

**Published:** 1896-06

**Authors:** 


*New Jersey.* One of the larger cities of northern New Jersey has been the centre of a hydrophobia scare, caused by the appearance of a rabid dog which succeeded in biting several persons before he was shot.

